# Validity and reliability of the kiddie schedule for affective disorders and schizophrenia present and lifetime version DSM-5 (K-SADS-PL-5) Spanish version

**DOI:** 10.1186/s12888-018-1773-0

**Published:** 2018-06-14

**Authors:** Francisco R. de la Peña, Lino R. Villavicencio, Juan D. Palacio, Fernando J. Félix, Marcela Larraguibel, Laura Viola, Silvia Ortiz, Marcos Rosetti, Andrea Abadi, Cecilia Montiel, Pablo A. Mayer, Sofía Fernández, Aurora Jaimes, Miriam Feria, Liz Sosa, Andrés Rodríguez, Patricia Zavaleta, Daniela Uribe, Frinne Galicia, Diana Botero, Santiago Estrada, Arturo F. Berber, Macarena Pi-Davanzo, Consuelo Aldunate, Gabriela Gómez, Ivannah Campodónico, Paula Tripicchio, Ignacio Gath, Manuel Hernández, Lino Palacios, Rosa E. Ulloa

**Affiliations:** 1 Adolescents’ Clinic, National Institute of Psychiatry Ramón de la Fuente Muñiz, Mexico City, Mexico; 2 Psychiatry Department, School of Medicine, Antioquia University, Medellin, Colombia; 3 Psychiatry Hospital Gustavo León Mojica, Aguascalientes, Mexico; 4 Psychiatry Universitary Clinic, School of Medicine, Autonomous University of Chile, Santiago de Chile, Chile; 5 Department of Pediatric Psychiatry, Childrens’ Hospital La Española, School of Medicine, Uruguay University, Montevideo, Uruguay; 6 National Autonomous University of Mexico, Mexico City, Mexico; 7 Institute of Cognitive and Translational Neuroscience, INECO Fundation, Favaloro University, Buenos Aires, Argentina; 8 Zulia University, Maracaibo, Venezuela; 9 Center for Postgraduate Studies, Latin University of Panama, Panama City, Panama; 10 Childrens’ Psychiatry Hospital Juan N. Navarro, Mexico City, Mexico

**Keywords:** K-SADS-PL, Validity, Reliability, DSM-5

## Abstract

**Background:**

There are various language adaptations of the Schedule for Affective Disorders and Schizophrenia for School Age Children Present and Lifetime Version (K-SADS-PL). In order to comply with the changes in DSM classification, the Spanish edition of the interview was in need of update and evaluation.

**Methods:**

K-SADS-PL was adapted to correspond to DSM-5 categories. All clinicians received training, and a 90% agreement was reached. Patients and their parents or guardians were interviewed and videotaped, and the videos were exchanged between raters. Factor analysis was performed and inter-rater reliability was calculated only in the case of diagnoses in which there were more than five patients.

**Results:**

A total of 74 subjects were included. The Factor Analysis yielded six factors (Depressive, Stress Hyperarousal, Disruptive Behavioral, Irritable Explosive, Obsessive Repetitive and Encopresis), representing 72% of the variance. Kappa values for inter-rater agreement were larger than 0.7 for over half of the disorders.

**Conclusions:**

The factor structure of diagnoses, made with the instrument was found to correspond to the DSM-5 disorder organization. The instrument showed good construct validity and inter-rater reliability, which makes it a useful tool for clinical research studies in children and adolescents.

**Electronic supplementary material:**

The online version of this article (10.1186/s12888-018-1773-0) contains supplementary material, which is available to authorized users.

## Background

Diagnostic clinical interviews are essential for child and adolescent psychiatric research, since they homogenize criteria and decrease sources of variability. The most commonly used semi-structured clinical interview is the Kiddie Schedule for Affective Disorders and Schizophrenia, Present and Lifetime version (K-SADS-PL) which is designed to correspond to the Diagnostic and Statistical Manual for Mental Disorders fourth edition (DSM-IV) [1].

There are several validity and reliability studies of different language adaptations of the K- SADS-PL. The original version was examined for its inter-rater, test-retest reliability and concurrent validity [2]. The Spanish version reported inter-rater reliability [3]. The Korean version reported inter-rater, test-retest reliability, consensus validity, and established its correlation with the Child Behavioral Check List (CBCL) [4]. The Icelandic version determined convergent and divergent validity in relation to the diagnosis of depression [5]. For the Iranian (Farsi) version, one study determined the validity by consensus as well as inter-rater and test-retest reliability [6], while a second study added concurrent validity of the instrument [7]. The Brazilian-Portuguese version determined the convergent validity with the CBCL [8]. Regarding the interview adaptations based on DSM-5 criteria, the Taiwanese team reported inter-rater reliability for the Mandarin K-SADS epidemiological version [9] while in a recent study, the Kenyan team found good test-retest reliability for the ADHD module of the K-SADS-PL [10].

Since the K-SADS-PL was designed to correspond to the DSM-IV, the instrument requires updating in order to include the new DSM-5 [11] categories. The organization of the categories was modified to include neurodevelopmental disorders. In addition, new diagnoses have been added and others have been modified. In particular, disruptive mood dysregulation disorder (DMDD), avoidant restrictive food intake disorder (ARFID) and binge eating disorder (BID) are new in DSM-5, while autism spectrum disorder (ASD), intermittent explosive disorder (IED) and social anxiety disorder (SAD) represent modified versions of diagnoses that were already in the DSM-IV. Clinical conditions not considered categories, such as non suicidal self injury (NSSI) and limited prosocial emotions (LPE) were also included. Since these changes may have modified the internal structure of the interview, a factor analysis of the K-SADS-PL-5 could help to determine how the diagnostic groups are integrated in the schedule.

A factor analysis allows us to construct a dimensional view of the diagnoses and permits a better understanding of the taxonomic commonalities and clinical comorbidities [12, 13]. Other validation methods such as convergent or divergent validity have failed to consider the possibility that “disorders might merge into one another with no natural boundary in between” [14]. Factor analysis of epidemiological data from structured clinical interviews has helped to identify factors explaining diagnostic groupings. Main factors have been termed as internalizing and externalizing: the former includes diagnoses of anxiety and depression, while the latter includes disruptive behaviors and substance use disorders [15, 16]. Recently, a study using a large, epidemiological sample of adolescents, has further divided these two factors into four: distress disorders, fear disorders, behavioral disorders, and substance disorders. The first two grouped within the internalizing factor, while the last two clustered within the externalizing factor [17]. Seeking further definition, the authors explored a fifth factor, which applied only to eating disorders [18]. It is worth noting that most studies use epidemiological samples and therefore studies in clinical settings could help to understand diagnostic grouping in such scenarios.

Inter-rater agreement is among the most important procedures for establishing reliability in regard to psychiatric interviews [19]. Several studies from different continents have included inter-rater reliability for the KSADS-PL [2–4, 6, 9]. In general, these studies suggest that inter-rater reliability in pediatric population are often larger for externalizing disorders than for internalizing disorders.

The objective of this study was to determine the construct validity and the inter-rater reliability of the K-SADS-PL-5 in an international multicenter study.

## Method

### Ethics

The present research was approved by “Comite de Ética en investigación del Instituto Nacional de Psiquiatría Ramón de la Fuente Múñiz” (CEI/C/003/2014) to be conducted in all sites. Written informed assent and consent were obtained from the parent or guardian and the child or adolescent.

### Subjects

The sample was comprised of children and adolescents (6 to 18 years old) who were referred to medical-psychiatric evaluation in any of the clinical sites. The parent or guardian and the child or adolescent were interviewed and videotaped for one to four sessions, each lasting 30 to 120 min.

### Sites

The sample was obtained from inpatient and outpatient services in four different Latin American countries. Four sites were located in Mexico (MX), three in Mexico City (MX1, MX2 and MX3) and one in Aguascalientes (MX4). The other three were located in South America, one in Medellin, Colombia (CO), another in Santiago de Chile, Chile (CL), and the last one in Montevideo, Uruguay (UR). Venezuela and Argentina only participated in the inter-rater evaluation.

### Description of the instrument

The K-SADS-PL-5 is a semi-structured diagnostic interview designed to collect information from the child or adolescent as well as their parents or other informants. A trained interviewer produces a better clinical estimate or summary for each symptom of all categories included. With this interview, it is possible to determine current episodes (defined as episodes that have occurred within the last six months) or past episodes. The current study used only information regarding current episodes. The K-SADS-PL-5 consists of a screening section and six supplements. Screening contains an introductory interview that covers the reason for consultation and general patient data as well as a screening section of the primary symptoms of each disorder. When at least one symptom is evaluated as definitive in the summary, the evaluation of the disorder is completed in the corresponding supplement. In this version, the six new or modified diagnoses, NSSI and LPE were included to be completed in the screening. The General Assessment Function Scale (GAF) and the World Health Organization Disability Assessment Scale (WHODAS) were also integrated into screening in order to harmonize with DSM-5. Supplement 1 includes depressive and bipolar disorders, supplement 2 includes psychotic disorders, supplement 3 includes anxiety, stress and obsessive compulsive disorders, supplement 4 includes disruptive behavior and impulse control disorders, supplement 5 includes substance use disorders and feeding and eating disorders, and the new supplement 6 includes neurodevelopmental disorders.

### Process

The first stage, carried out from January 2014 to June 2015 included the following steps, (i) a Latin American international clinical team comprising the current authors reviewed and adapted the K-SADS-PL Spanish version [3] to the DSM-5 structure followed by (ii) the inclusion of the six new or modified diagnoses under consensus and (iii) the text review and testing with clinicians, patients and parents in order to obtain a final version incorporating all comments and suggestions. The second stage, carried out from August 2015 to January 2016, consisted of 20 h workshops to train interviewers. A consensus criteria of > 90% match between trainer and trainees’ diagnoses was reached, as done in previous studies [9, 19]. Workshops were held in Buenos Aires, Medellin, and Mexico City. During the third stage, carried out from February to July 2016, clinicians from each site sought to interview 10 subjects (five children and five adolescents); if this quota was not met, the number of patients was compensated by sites with greater capacity. This number of patients was chosen a priori in order to balance the number of children and adolescents from each site. In every case, informed assent and consent was obtained previous to the interviews. Each of the sites was coordinated by a child psychiatrist with more than fifteen years of clinical experience. The rest of the raters were child psychiatrists and clinical psychologists. Each site coordinator maintained supervision of the interviews and communication with the principal investigator with whom any doubt was resolved. The fourth stage, carried out from August 2016 to February 2017, consisted in the inter-site exchange and rating of the videotaped interviews, which was done every three weeks through a privately shared video system over the internet. The principal investigator randomly assigned every rated interview to at least two raters. Every rater evaluated the interview using the K-SADS-PL-5 and sent back the results to the principal investigator. This resulted in three separate evaluations for each interview.

### Statistical analysis

The general demographic and diagnostic data of the subjects was described using percentages, medians and interquartile ranges. For the factor analysis, a polychoric correlation was performed on a dichotomous matrix of diagnoses obtained from the summary section of the K-SADS-PL-5 (classified as either present or absent) discarding those that were completely absent or had low prevalence (tic disorder, cyclothymia, bipolar disorder, enuresis, anorexia disorder, bulimia disorder, avoidant restrictive food intake disorder, tobacco use disorder, substance use disorder schizophrenia). Using a Cattell’s scree test, the resulting number of factors and the corresponding values were plotted. With this criteria, we settled on six factors and proceeded to perform a factor analysis using the generalized least squares method with an oblimin rotation, as recommended by Stuart [20]. We established minimal factorial load for values > 0.40 and focused on the maximum loading per factor. Cattell’s scree test was performed using the *scree* function, the polychoric correlation was performed using the *cor.ci* function and the factor analysis was performed using the *fa* function; all functions belong to the psych package [21]. For inter-rater reliability, Cohen’s Kappa tests were calculated for diagnoses with 5 or more subjects [22]. All analyses were performed in R [23].

## Results

### Sample characteristics

Eighty children and adolescents from all venues were evaluated; six were excluded because they did not have complete screening or supplements. Results are shown for 74 subjects. Mexico contributed with 50 from four sites: MX1 = 30, MX2 = 10, MX3 = 8 and MX4 = 2; South America contributed 24 from three sites: CO = 15, CL = 5 and UR = 4. The sample were 59% males, 62.1% children, the median age and interquartile range (IQR) was 11 (9–14) years. They presented a median number of comorbid diagnoses per patient of 3 (1–5.75). Complete demographic and diagnostic clinical characteristics of the sample can be reviewed in a previous report [24].

### Factor analysis

The Factor Analysis of the interview revealed six factors that explained 72% of the total variance. In addition, a five (64% of variance explained) and a seven (75% of variance explained) factor models were performed. We found that three factors, Depressive Factor (DF), Disruptive Behavior Factor (DBF) and an Irritable Explosive Factor (IEF) had high stability and were present in all solutions. The other three in the six factor solution were Stress Hyperarousal (SHF), Obsessive Repetitive (ORF) and Encopresis Factors (EnF). The eigenvalues for each factor and the factor load for the six factor solution are shown in Table 1. The five and seven factor solutions can be seen in the Additional file 1: Tables S1 and S2.

**Table 1:** Factor analysis on K-SADS-PL-5 diagnoses with six factors

		Factors					
		DF (6.48)	SHF (3.16)	DBF (2.4)	IEF (1.59)	ORF (1.48)	EnF (1.26)
	Prop. Of Variance Explained	16%	16%	12%	11%	11%	6%
*Dx*	*Non Suicidal Self Injuries*	**0.98**	0.05	−0.01	− 0.14	− 0.03	− 0.07
	*Selective Mutism*	**0.76**	0.01	0.18	0.07	0.11	0.06
	*Disruptive Mood Dysregulation Disorder*	**0.56**	−0.34	0.05	0.39	0.03	0.06
	*Dysthymic Disorder*	**0.54**	0.35	−0.06	−0.17	0.1	0.33
	*Major Depressive Disorder*	**0.47**	0.29	−0.06	0.07	0.41	−0.56
	*Separation Anxiety Disorder*	0.41	0.09	−0.34	**0.62**	−0.28	0.15
	*Generalized Anxiety Disorder*	0.37	0.3	−0.19	0.34	0.01	0.14
	*Agoraphobia*	0.34	0.38	0.06	0.12	0.31	−0.09
	*Oppositional Defiant Disorder*	0.28	−0.1	0.46	**0.61**	−0.01	−0.19
	*Encopresis*	0.21	0.22	0.15	0.17	0.24	**0.56**
	*Social Anxiety Disorder*	0.2	**0.6**	0.26	−0.12	−0.17	−0.09
	*Attention Deficit Hyperactivity Disorder*	0.17	−0.13	**0.49**	0.19	−0.41	0
	*Autism Spectrum Disorders*	0.08	0.04	0.13	−0.16	**0.55**	0.53
	*Binge Eating Disorder*	0.04	**0.58**	−0.22	0.22	0.37	0.11
	*Limited Prosocial Emotions*	0.04	0.02	**0.99**	−0.05	0.1	0.01
	*Obsessive Compulsive Disorder*	0.01	−0.02	0.05	0.11	**0.97**	0
	*Panic Disorder*	−0.02	**1**	0.01	−0.05	0.01	0
	*Post Traumatic Stress Disorder*	−0.06	**0.65**	0.05	0.24	−0.18	0.03
	*Conduct Disorder*	−0.09	0.15	**0.71**	0.28	−0.08	0.2
	*Specific Phobia*	−0.11	**0.44**	0.14	−0.04	−0.01	− 0.05
	*Intermittent Explosive Disorder*	−0.27	0.05	0.17	**0.9**	0.23	−0.01

Factor name (eigenvalue). Abbreviation: *DF* Depressive Factor, *SHF* Stress Hyperarousal Factor, *DBF* Disruptive Behavior Factor, *IEF* Irritable Explosive Factor, *ORF* Obsessive Repetitive Factor and *EnF* Encopresis Factor

Values in bold show highest loadings and indicate which diagnoses compose each factor

### Inter-rater reliability

The Kappa correlation coefficients were established for 11 categories, NSSI and, LPE. The agreement between the disruptive behavior disorders was greater than that of anxiety and depressive disorders. Details are shown in Table 2.

**Table 2:** Kappa Correlations for each disorder

Dx	*n*	Kappa	95% Conf. Int.
*Major Depressive Disorder*	21	0.77	0.63–0.91
*Disruptive Mood Dysregulation Disorder*	18	0.53	0.34–0.71
*General Anxiety Disorder*	14	0.70	0.52–0.88
*Separation Anxiety Disorder*	18	0.44	0.22–0.66
*Social Anxiety Disorder*	20	0.64	0.48–0.81
*Specific Phobia*	18	0.73	0.57–0.90
*Post Traumatic Stress Disorder*	7	0.39	0.09–0.69
*Attention Deficit Hyperactivity Disorder*	43	0.92	0.84–1.00
*Oppositional and Defiant Disorder*	30	0.80	0.69–0.92
*Conduct Disorder*	5	0.78	0.55–1.00
*Intermittent Explosive Disorder*	19	0.67	0.50–0.83
*Limited Prosocial Emotions*	9	0.29	0.03–0.55
*Non Suicidal Self injuries*	8	0.46	0.20–0.73

## Discussion

The main goal of current study was to establish construct validity and inter-rater reliability for the Spanish version of K-SADS-PL-5. A factor analysis revealed six groups of interest and reliability analysis showed Kappa values > 0.7 in the majority of the diagnostic categories.

We obtained a factor solution suggesting robust diagnostic groupings. Below, we discuss each factor independently.

The DF included major depressive disorder (MDD), NSSI, selective mutism (SM), DMDD and dysthimic disorder. Even though NSSI is not a mood disorder per se, it is a phenomenon frequently related to affective disorders [25, 26]. DMDD is a new disorder in the affective disorders chapter, and it has been shown in follow up studies to be associated with depression and anxiety [27]. The inclusion of SM in this factor could reflect its frequent comorbidity with depressive disorders [28].

The SHF included SAD, BED, post traumatic stress disorder (PTSD), panic disorder (PD) and specific phobia (SPH). Hyperarousal in social situations is a physiological characteristic that has been shown as a risk factor for later childhood symptoms of social anxiety and it has been proposed as a biological mechanism in the intergenerational transmission of SAD [29]. The relationship between anxiety disorders and BED has been demonstrated in adolescent clinical samples [30]. Previous evidence mentions stressful life events in early childhood as predictors of PD and SPH [31] and to be associated with BED [32]; a history of symptoms of PTSD also predict the later onset of binges [33]. Essentially, the evidence shows that stress life events may represent the main axis of the SHF.

The DBF included attention deficit hyperactivity disorder (ADHD), conduct disorder (CD) and LPE. These disorders are frequently comorbid [34]. A meta-analysis found CD in up to 41% of individuals with ADHD [35]. Up to one third of children with CD manifest LPE specifier symptoms [36]. These disorders are known to increment the global burden of disease and have an important implication in the functioning and wellbeing of individuals [37]. Several researchers have tried to study the interaction between them, for example, some studies have demonstrated that boys and girls with CD and meeting criteria for the LPE specifier showed more impairment in psychosocial areas [38–40]. In ADHD, impairment and comorbidity with CD may be moderated by LPE characteristics, especially in delinquent [41] and antisocial behavior [42]. The association between ADHD, CD and LPE also impacts and moderates psychosocial impairment among young adults [43].

The IEF included separation anxiety disorder (SAND), oppositional defiant disorder (ODD) and IED. SAND and ODD were associated in general population [44], but there is a lack of information about IED in association with ODD and SAND. Interestingly SAND and ODD present a bi-factorial load: ODD was present in the IEF (0.61) and in the DBF (0.46). Previous reports had described the integration of ODD symptoms grouping with emotional lability and irritability and not only with disruptive behaviors [45]. Furthermore, SAND was present in the IEF (0.62) and the DF (0.41), according to other researchers SAND may fit in irritability and anxiety dimensions [46]. The biggest load in the IEF was for IED (0.9) and did not load into any other factor, which suggests that the characteristics of IED represent the main characteristics of this factor.

The ORF grouped Obsessive Compulsive Disorder (OCD) and ASD. This highlights the repetitive component in these disorders [47]. Finally, the fact that encopresis constituted a single factor, could be related to its complex clinical characteristics and comorbidity [48, 49]. This is further supported by the results of the five and seven factor analysis, in which it loaded on a miscellaneous factor in the former and did not load into any factor on the latter.

The results of the current report correspond, at least in part, to previous reports describing epidemiological samples [15–17], particularly in regard to the integration of internalizing disorders as those included in the DF and SHF, as well as in the externalizing disorders, as shown in the DBF, IEF.

Inter-rater reliability was high for CD, ADHD and ODD, but low for NSSI, LPE and PTSD. In previous K-SADS-PL studies, the highest inter-rater agreement was for CD [3, 4, 6]. Clinical presentation of CD comprises behaviors that are usually easy for most people to recognize. High agreement between raters for ADHD and ODD had also been reported [3, 4, 6]. These results are of relevance since these disorders are among the most common reason for psychiatric consultation in pediatric population. The lowest Kappa values were for LPE (0.29) and PTSD (0.39). Curiously, the interview format for these two disorders do not strictly follow the standard interview format of the K-SADS-PL. The diagnosis of PTSD requires two subsequent different skip criteria, while LPE involves a criterium based on four items. In terms of impact on clinical practice, these aspects may suggest that (i) the instrument format for these two clinical conditions could be improved and (ii) that in order to increase reliability, more accurate training may be needed.

Interestingly, our study reports Kappa values above 0.7 for more than 50% of the disorders which contrasts with the Taiwanese study [9] where inter-rater agreement never reached values over 0.7 even though both studies established a 90% agreement in their training procedures. This could be explained by several factors: (i) the clinical versus the school-based sample, (ii) the more severe symptomatology and (iii) easier recognition in the clinical population.

Some study limitations should be considered. Although it is a Latin American multi-center study, not all the centers provided an equal number of cases which limited hemispheric representation. The sample size is small relative to the number of variables included in the factor analysis, this was due to the comprehensive nature of K-SADS-PL, which comprises a large number of diagnostic categories (*N* = 43). Sample sizes in this range can be seen in other studies evaluating the psychometric properties of the interview [4, 5, 8, 10]. To somewhat reduce this problem, we removed disorders with a low prevalence from the factor analysis. Furthermoe, the relatively small number of cases with some DSM-5 diagnosis only allowed to established 13 Kappa values, although it surpasses diagnoses number in previous Spanish K-SADS-PL version.

## Conclusion

Application of the K-SADS-PL-5 Spanish version, which incorporates changes in the DSM-5, yielded factors that showed coherence with DSM-5 diagnostic groups. Also, a good inter-rater reliability was obtained for major disorders. All these elements make it a useful tool for clinical research studies in children and adolescents.

## Additional file

Additional file 1: **Table S1.** Factor analysis on K-SADS-PL-5 diagnoses using 5 factors. Factor name (eigenvalue). Abbreviation: Miscellaneous Factor (MF), Depressive Factor (DF), Disruptive Behavior Factor (DBF), Irritable-Explosive Factor (IEF) and Separation Anxiety Disorder Factor (SADF). **Table S2.** Factor analysis on K-SADS-PL-5 diagnoses using 7 factors. Factor name (eigenvalue). Abbreviation: Miscellaneous Factor (MF), Depressive Factor (DF), Disruptive Behavior Factor (DBF), Irritable Explosive Factor (IEF), Separation Anxiety Disorder Factor (SADF), Phobic Factor (PhF) and Depressive Obsessive Factor (DOF). (DOC 80 kb)

## Abbreviations

ADHD: Attention Deficit Hyperactivity Disorder
ARFID: Avoidant Restrictive Food Intake Disorder
ASD: Autism Spectrum Disorder
BED: Binge Eating Disorder
CBCL: Child Behavioral Check List
CD: Conduct Disorder
CL: Chile
CO: Colombia
DBF: Disruptive Behavior Factor
DF: Depressive Factor
DMDD: Disruptive Mood Dysregulation Disorder
DSM-5: Diagnostic and Statistical Manual for Mental Disorders fifth edition
DSM-IV: Diagnostic and Statistical Manual for Mental Disorders fourth edition
EnF: Encopresis Factor
GAF: General Assessment Function Scale
IED: Intermittent Explosive Disorder
IEF: Irritable Explosive Factor
IQR: Inter Quartile Range
K-SADS-PL: Schedule for Affective Disorders and Schizophrenia for School Age Children Present and Life Time Version
K-SADS-PL-5: Schedule for Affective Disorders and Schizophrenia for School Age Children Present and Life Time Version DSM-5
LPE: Limited Prosocial Emotions
MDD: Major Depressive Disorder
MX: Mexico
NSSI: Non Suicidal Self Injury
OCD: Obsessive Compulsive Disorder
ODD: Oppositional Defiant Disorder
ORF: Obsessive Repetitive Factor
PD: Panic Disorder
PTSD: Post Traumatic Stress Disorder
SAD: Social Anxiety Disorder
SAND: Separation Anxiety Disorder
SHF: Stress Hyperarousal Factor
SM: Selective Mutism
SPH: Specific Phobia
UR: Uruguay
WHODAS: World Health Organization Disability Assessment Scale
