# Supplementary material for: Validity and reliability of the kiddie schedule for affective disorders and schizophrenia present and lifetime version DSM-5 (K-SADS-PL-5) Spanish version
Source: BMC Psychiatry. 2018 Jun 14;18:193. doi: 10.1186/s12888-018-1773-0 (PMC6001018; doi:10.1186/s12888-018-1773-0)
Supplement: Supplementary file 1 — Table S1. Factor analysis on K-SADS-PL-5 diagnoses using 5 factors. Factor name (eigenvalue). Abbreviation: Miscellaneous Factor (MF), Depressive Factor (DF), Disruptive Behavior Factor (DBF), Irritable-Explosive Factor (IEF) and Separation Anxiety Disorder Factor (SADF). Table S2. Factor analysis on K-SADS-PL-5 diagnoses using 7 factors. Factor name (eigenvalue). Abbreviation: Miscellaneous Factor (MF), Depressive Factor (DF), Disruptive Behavior Factor (DBF), Irritable Explosive Factor (IEF), Separation Anxiety Disorder Factor (SADF), Phobic Factor (PhF) and Depressive Obsessive Factor (DOF). (DOC 80 kb) [file 12888_2018_1773_MOESM1_ESM.doc]

**Table S1.** Factor analysis on K-SADS-PL-5 diagnoses using 5 factors. Factor name (eigenvalue). Abbreviation: Miscellaneous Factor (MF), Depressive Factor (DF), Disruptive Behavior Factor (DBF), Irritable-Explosive Factor (IEF) and Separation Anxiety Disorder Factor (SADF).

|  |  | Factors |  |  |  |  |
| --- | --- | --- | --- | --- | --- | --- |
|  |  | MF  (6.48) | DF  (3.16) | DBF  (2.4) | IEF  (1.59) | SADF (1.48) |
|  | Prop. Of Variance Explained | 19% | 15% | 11% | 10% | 9% |
| *Dx* | *Agoraphobia* | 0.74 | 0.2 | 0.1 | 0.02 | 0.14 |
|  | *Panic Disorder* | 0.72 | 0.12 | -0.04 | -0.06 | 0.01 |
|  | *Binge Eating Disorder* | 0.69 | 0.11 | -0.22 | 0.25 | 0.04 |
|  | *Obsessive Compulsive Disorder* | 0.63 | 0.11 | 0.07 | 0.38 | -0.37 |
|  | *Dysthymic Disorder* | 0.57 | 0.27 | 0.04 | -0.34 | 0.27 |
|  | *Encopresis* | 0.57 | -0.12 | 0.32 | 0.02 | 0.39 |
|  | *Autism Spectrum Disorders* | 0.56 | -0.07 | 0.21 | -0.03 | -0.18 |
|  | *Specific Phobia* | 0.42 | -0.16 | 0.19 | -0.12 | 0.18 |
|  | *Major Depressive Disorder* | 0.38 | 0.69 | -0.15 | 0.14 | -0.19 |
|  | *Generalized Anxiety Disorder* | 0.37 | 0.33 | -0.18 | 0.18 | 0.28 |
|  | *Post Traumatic Stress Disorder* | 0.28 | 0.01 | 0.06 | 0.1 | 0.35 |
|  | *Social Anxiety Disorder* | 0.24 | 0.43 | 0.18 | -0.16 | 0.02 |
|  | *Intermittent Explosive Disorder* | 0.15 | -0.15 | 0.14 | 0.91 | 0.18 |
|  | *Selective Mutism* | 0.14 | 0.7 | 0.19 | -0.05 | 0.14 |
|  | *Non Suicidal Self Injuries* | 0.13 | 0.83 | 0 | -0.32 | 0.13 |
|  | *Conduct Disorder* | 0.12 | -0.13 | 0.68 | 0.29 | -0.01 |
|  | *Separation Anxiety Disorder* | 0.07 | 0.13 | -0.19 | 0.18 | 0.91 |
|  | *Limited Prosocial Emotions* | 0.06 | 0.1 | 0.95 | 0.04 | -0.2 |
|  | *Oppositional Defiant Disorder* | -0.26 | 0.51 | 0.37 | 0.58 | 0.1 |
|  | *Attention Deficit Hyperactivity Disorder* | -0.28 | -0.02 | 0.55 | -0.05 | 0.4 |
|  | *Disruptive Mood Dysregulation Disorder* | -0.32 | 0.6 | 0.04 | 0.3 | 0.19 |

**Table S2.** Factor analysis on K-SADS-PL-5 diagnoses using 7 factors. Factor name (eigenvalue). Abbreviation: Miscellaneous Factor (MF), Depressive Factor (DF), Disruptive Behavior Factor (DBF), Irritable Explosive Factor (IEF), Separation Anxiety Disorder Factor (SADF), Phobic Factor (PhF) and Depressive Obsessive Factor (DOF).

|  |  | MF  (6.48) | DF  (3.16) | DBF  (2.4) | IEF  (1.59) | SAD  (1.48) | PhF  (1.26) | DOF  (1.08) |
| --- | --- | --- | --- | --- | --- | --- | --- | --- |
|  | Prop. Of Variance Explained | 14% | 13% | 12% | 12% | 9% | 9% | 6% |
| *Dx* | *Binge Eating Disorder* | 0.99 | -0.01 | -0.09 | 0.11 | 0.02 | -0.05 | 0.12 |
|  | *Panic Disorder* | 0.66 | -0.02 | -0.01 | -0.15 | 0.38 | 0.12 | 0.12 |
|  | *Autism Spectrum Disorders* | 0.6 | 0.01 | 0.36 | -0.2 | -0.04 | 0.05 | -0.2 |
|  | *Dysthymic Disorder* | 0.46 | 0.51 | 0.03 | -0.24 | 0.1 | 0.17 | -0.2 |
|  | *Generalized Anxiety Disorder* | 0.45 | 0.2 | -0.2 | 0.33 | 0.31 | 0.15 | -0.2 |
|  | *Obsessive Compulsive Disorder* | 0.39 | -0.1 | 0.24 | 0.11 | -0.19 | 0.31 | 0.43 |
|  | *Encopresis* | 0.31 | 0.23 | 0.3 | 0.1 | -0.19 | 0.34 | -0.31 |
|  | *Post Traumatic Stress Disorder* | 0.28 | -0.09 | -0.04 | 0.19 | 0.34 | 0 | 0 |
|  | *Major Depressive Disorder* | 0.2 | 0.4 | -0.09 | 0.09 | 0.03 | 0.2 | 0.72 |
|  | *Conduct Disorder* | 0.13 | 0.01 | 0.75 | 0.24 | -0.16 | 0.03 | -0.18 |
|  | *Intermittent Explosive Disorder* | 0.13 | -0.32 | 0.17 | 0.87 | -0.1 | 0.2 | 0.05 |
|  | *Social Anxiety Disorder* | 0.08 | 0 | 0.07 | 0 | 0.95 | 0.08 | -0.01 |
|  | *Separation Anxiety Disorder* | 0.05 | 0.33 | -0.4 | 0.59 | 0 | 0.19 | -0.29 |
|  | *Non Suicidal Self Injuries* | 0.01 | 0.97 | -0.01 | -0.11 | 0.01 | 0.07 | 0.13 |
|  | *Agoraphobia* | 0 | 0.21 | 0.03 | 0.06 | 0.14 | 0.84 | 0.14 |
|  | *Selective Mutism* | -0.02 | 0.69 | 0.17 | 0.17 | 0.1 | 0.13 | 0.06 |
|  | *Oppositional Defiant Disorder* | -0.07 | 0.21 | 0.37 | 0.74 | 0.16 | -0.2 | 0.18 |
|  | *Attention Deficit Hyperactivity Disorder* | -0.08 | 0.17 | 0.44 | 0.18 | -0.06 | -0.13 | -0.2 |
|  | *Limited Prosocial Emotions* | -0.1 | 0.03 | 0.97 | 0.02 | 0.14 | 0.05 | 0.06 |
|  | *Disruptive Mood Dysregulation Disorder* | -0.11 | 0.46 | 0.03 | 0.5 | 0.18 | -0.31 | 0 |
|  | *Specific Phobia* | -0.18 | -0.21 | 0.03 | -0.05 | 0.41 | 0.56 | -0.01 |
